# The process of developing a community-based research agenda with lesbian, gay, bisexual, transgender and queer youth in the Northwest Territories, Canada

**DOI:** 10.3402/ijch.v74.28188

**Published:** 2015-08-24

**Authors:** Carmen H. Logie, Candice Lys

**Affiliations:** 1 Factor-Inwentash Faculty of Social Work, University of Toronto, Toronto, ON, Canada; 2 Women's College Research Institute, Women's College Hospital, University of Toronto, Toronto, ON, Canada; 3 Dalla Lana School of Public Health, University of Toronto, Toronto, ON, Canada; 4 Fostering Open eXpression Among Youth (FOXY), Yellowknife, NWT, Canada

**Keywords:** Arctic, youth, lesbian, gay, bisexual, transgender

## Abstract

**Background:**

Youth in Canada's Northwest Territories (NWT) experience sexual and mental health disparities. Higher rates of sexual and mental health concerns among lesbian, gay, bisexual, transgender and queer (LGBTQ) youth in comparison with heterosexual and cisgender peers have been associated with stigma and discrimination. Although LGBTQ youth in the NWT are situated at the nexus of Northern and LGBTQ health disparities, there is little known about their health, well-being and experiences of stigma. This short communication discusses the process of developing a LGBTQ youth community-based research programme in the NWT.

**Methods:**

We developed an interdisciplinary research team of LGBTQ and allied young adults, including indigenous and non-indigenous researchers, community organisers and service providers in the NWT. We conducted meetings in Yellowknife with LGBTQ youth (*n=*12) and key stakeholders (*n*=15), including faculty, students, community groups and health and social service providers. Both meetings included LGBTQ and allied participants who were LGBTQ, indigenous, youth and persons at the intersection of these identities.

**Results:**

LGBTQ youth participants discussed community norms that devalued same sex identities and stigma surrounding LGBTQ-specific services and agencies. Stigma among LGBT youth was exacerbated for youth in secondary schools, gender non-conforming and transgender youth and young gay men. In the stakeholder meeting, service providers discussed the importance of integrating LGBTQ issues in youth programmes, and LGBTQ community groups expressed the need for flexibility in service delivery to LGBTQ youth. Stakeholders identified the need to better understand the needs of indigenous LGBTQ youth in the NWT.

**Conclusions:**

Community-based LGBTQ groups, researchers and health and social service providers are interested in addressing LGBTQ youth issues in the NWT. The emergence of LGBTQ community building, support groups and activism in Northern Canada suggests that this is an opportune time to explore LGBTQ youth health.

Health disparities among youth in Canada's Northwest Territories (NWT) are a pressing concern. In the NWT, youth suicide rates are twice the national average with rates for smaller, rural communities double that of the capital, Yellowknife (1). Reported sexually transmitted infections rates, such as chlamydia, in the NWT (2,086/100,000) are nearly 10 times the national average (278/100,000) (2). Stigma and discrimination, targeting lesbian, gay, bisexual, transgender and queer (LGBTQ) people, contribute to sexual and mental health disparities in comparison with their heterosexual and cisgender peers. For example, stigmatisation of LGBTQ people in healthcare settings operates as a distal structural driver of HIV by limiting access to sexual health care and HIV prevention and testing. The impacts are profound: gay, bisexual and other men who have sex with men over 15 years old comprised almost half (46.6%) of new HIV infections in Canada in 2011, while only accounting for 2.6% of the population (3). Scant research has addressed experiences of stigma and health among LGBTQ youth in the NWT. Our objective is to discuss the process of developing a LGBTQ youth community-based research programme in the NWT.

## Methods

This research was initiated by LGBTQ community leaders in Yellowknife, and an indigenous organisation, Fostering Open eXpression Among Youth (FOXY), in the NWT focused on young women's sexual health. We established an interdisciplinary team comprising LGBTQ and allied youth and adults in the NWT, including researchers, community organisers and service providers. Second, we held a meeting with LGBTQ youth (*n*=12), recruited using convenience sampling, in Yellowknife during NWT Pride (August 2014) where we discussed issues of stigma, health and social support among LGBTQ youth in the NWT. Third, we held a meeting with key stakeholders (*n*=15) from Yellowknife and Hay River interested in promoting LGBTQ youth health, including college faculty, students, LGBT community groups, social service providers, sexual healthcare programme staff and LGBTQ youth in March 2015. Stakeholders were purposively selected to reflect the diversity of the NWT and the LGBTQ communities. Stakeholders included people who were indigenous, LGBTQ, youth, transgender, gender non-conforming and people at the intersection of these identities.

## Findings

In the meeting, LGBTQ youth discussed community norms that devalued same sex identities and stigma surrounding LGBTQ-specific services. Yet, they described that there were few instances of violence and enacted stigma due to small, close-knit communities. Stigma was exacerbated for LGBTQ youth in secondary schools, particularly among gender non-conforming and transgender youth, and those in smaller communities. Transgender and gender non-conforming participants also highlighted a lack of role models that contributed to feelings of isolation. While recent activism (e.g. NWT Pride) generated media and community discussion of LGBTQ issues, many youth discussed invisibility in school and health care. In the stakeholder meeting, service providers discussed the importance of integrating LGBTQ issues and discussions in youth programmes, particularly in rural regions. LGBTQ community groups expressed the need for flexibility in the ways services are delivered to LGBTQ youth, citing the use of online support services (e.g. web-based) and social media (e.g. Tumblr) as places LGBTQ youth in the NWT access support. Stakeholders also articulated the need to enhance the visibility of diverse (e.g. age, ethnicity, gender expression and sexuality) LGBTQ people from, and living in, the NWT to reduce the feelings of isolation. Stakeholders identified the need to better understand the needs of indigenous LGBTQ youth in the NWT who may not be accessing mainstream LGBTQ-specific community groups or events to the same degree as non-indigenous LGBTQ youth.

## Conclusions

Key informants revealed interest and willingness of community-based LGBTQ health, education and social programmes to address LGBTQ youth issues in the NWT. Our report has limitations. We did not collect empirical data and therefore cannot represent in-depth perspectives of LGBTQ youth experiences in the NWT. Instead, we aim to provide a snapshot of the process of building this research programme through engaging allied, LGBTQ and indigenous youth and service providers. Future research should also involve elders and other indigenous community leaders, and people from various regions in the NWT. Participants discussed not only the importance of LGBTQ-specific services for youth, but also the need to integrate sexual and gender diversity into health, education and social programmes (e.g. training healthcare providers about LGBTQ issues; forming gay-straight alliances in schools). Additional research with LGBTQ youth in the NWT should explore (a) intersections of identities (e.g. sexuality, ethnicity, gender, rural/urban and indigenous identities); (b) health priorities; and (c) stigma and healthcare access. There has been an emergence of LGBTQ community building, support groups and activism in the NWT – such as NWT Pride, Hay River Pride and It Gets Better Yellowknife – suggesting this is an opportune time to explore LGBTQ youth health.
